# Kinetic studies and predictions on the hydrolysis and aminolysis of esters of 2-*S*-phosphorylacetates

**DOI:** 10.3762/bjoc.6.87

**Published:** 2010-08-16

**Authors:** Milena Trmčić, David R W Hodgson

**Affiliations:** 1 Centre for Bioactive Chemistry, Department of Chemistry, Durham University, Science Laboratories, South Road, Durham DH1 3LE, United Kingdom

**Keywords:** aminolysis, heterobifunctional cross-linker, hydrolysis, kinetics, thiophosphate

## Abstract

**Background:** Heterobifunctional cross-linking agents are useful in both protein science and organic synthesis. Aminolysis of reactive esters in aqueous systems is often used in bioconjugation chemistry, but it must compete against hydrolysis processes. Here we study the kinetics of aminolysis and hydrolysis of 2-*S*-phosphorylacetate ester intermediates that result from displacement of bromide by a thiophosphate nucleophile from commonly used bromoacetate ester cross-linking agents.

**Results:** We found cross-linking between uridine-5′-monophosphorothioate and D-glucosamine using *N*-hydroxybenzotriazole and *N*-hydroxysuccinimde bromoacetates to be ineffective. In order to gain insight into these shortfalls, 2-*S*-(5′-thiophosphoryluridine)acetic acid esters were prepared using *p*-nitrophenyl bromoacetate or *m*-nitrophenyl bromoacetate in combination with uridine-5′-monophosphorothioate. Kinetics of hydrolysis and aminolysis of the resulting *p*- and *m*-nitrophenyl 2-*S*-(5′-thiophosphoryluridine)acetates were determined by monitoring the formation of phenolate ions spectrophotometrically as a function of pH. The *p*- and *m*-nitrophenyl 2-*S*-(5′-thiophosphoryluridine)acetates showed similar reactivity profiles despite the significant difference in the p*K*_aH_ values of their nitrophenolate leaving groups. Both were more reactive with respect to hydrolysis and aminolysis in comparison to their simple acetate progenitors, but their calculated selectivity towards aminolysis vs hydrolysis, while reasonable, would not lead to clean reactions that do not require purification. Extrapolations of the kinetic data were used to predict leaving group p*K*_a_ values that could lead to improved selectivity towards aminolysis while retaining reasonable reaction times.

**Conclusions:** Both *p*- and *m*-nitrophenyl 2-*S*-(5′-thiophosphoryluridine)acetates show some selectivity towards aminolysis over hydrolysis, with the *m*-nitrophenolate system displaying slightly better selectivity. Extrapolation of the data for hydrolysis and aminolysis of these esters suggests that the use of readily accessible trifluoroethyl 2-*S*-(5′-thiophosphoryluridine)acetate with a leaving group p*K*_aH_ of 12.4 should afford better selectivity while maintaining reasonable reaction times. Kinetically, *p*- and *m*-nitrophenyl 2-*S*-(5′-thiophosphoryluridine)acetates show similar properties to *o*-nitrophenyl 2-*S*-ethylacetate, and show no evidence for intramolecular catalysis of hydrolysis or aminolysis by the phosphoryl groups.

## Introduction

**Scheme 1:**
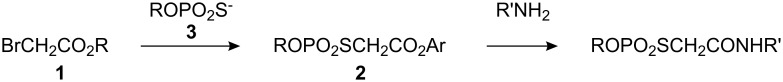
Use of 2-bromoacetic acid esters as heterobifunctional cross-linking agents.

Heterobifunctional cross-linking agents are used widely in protein science for forming covalently-bonded protein-protein complexes [1] and protein-small molecule systems [2]. *S*-Alkylation and *N*-acylation processes are used together extensively as orthogonal methods to effect hetero-cross-linking. *S*-Alkylation processes are not affected by competing hydrolysis, however, *N*-acylation using reactive esters is hampered by competing hydrolysis processes. The aminolysis and hydrolysis of activated esters has been well studied [3], however, the hydrolysis and aminolysis kinetics of 2-*S*-phosphorylacetic acid esters **2**, which are present as intermediates when using 2-bromoacetic acid esters **1** as heterobifunctional cross-linking agents with thiophosphate systems **3** (Scheme 1), have not been investigated.

In this paper we present our findings into the use of bromoacetate-based cross-linking agents with thiophosphate nucleophiles **3**, where our aim was to generate nucleoside-pyrophosphate mimics as potential glycosyl transferase inhibitors under aqueous conditions. Initially, we focus on the use of the bromoacetic acid esters of *N*-hydroxybenzotriazole **1** (R = Bt) and *N*-hydroxysuccinimide **1** (R = NHS) before moving on to studies on the aminolysis and hydrolysis kinetics of *p*- and *m*-nitrophenyl 2-*S*-(5′-thiophosphoryluridine)acetates **7** (R = *p*NP) and **7** (R = *m*NP) with a view towards tuning the leaving group properties of 2-*S*-phosphorylacetic acid esters **2** in such a way as to favour aminolysis over undesired hydrolysis processes. In addition to kinetic studies, we present predictions of leaving group characteristics, based on correlation with literature data, that should allow for improvement in the performance of 2-bromoacetic acid esters **1** as heterobifunctional cross-linking agents, particularly in the context of small-molecule synthetic applications.

## Results and Discussion

**Scheme 2:**
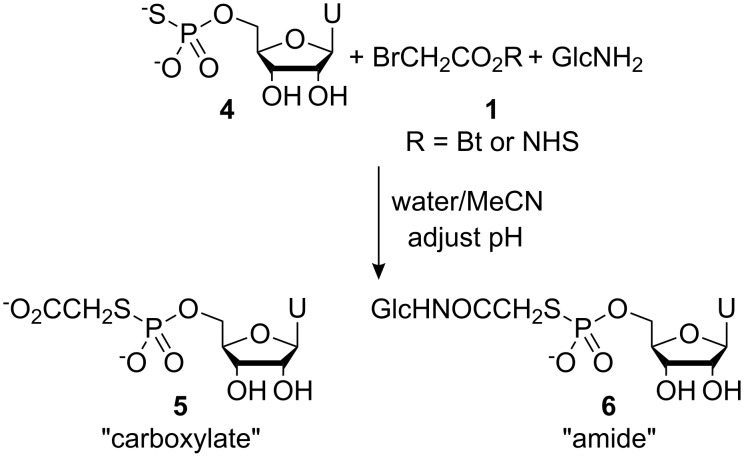
Cross-linking between thiophosphate **4**, D-glucosamine (GlcNH_2_) and bromoacetyl-*N*-hydroxybenzotriazole **1** (R = Bt) or bromoacetyl-*N*-hydroxysuccinimide **1(** R = NHS) in water/MeCN.

2-Bromoacetic acid esters **1** were prepared by allowing bromoacetyl bromide to react with the respective *N*-hydroxy species or nitrophenols in the presence of pyridine. After standard aqueous work-up, the products were 70–99% pure as measured by integration of signals in ^1^H NMR spectra, and were used without further purification. Uridine-5′-monophosphorothioate **4** was prepared using an adaptation of Whitesides’ procedure for the thiophosphorylation of adenosine [4]. We performed preliminary studies into thiophosphate-amine cross-linking using both *N*-hydroxybenzotriazole **1** (R = Bt) and *N*-hydroxysuccinimde **1** (R = NHS) esters of bromoacetic acid with thiophosphate **4** and D-glucosamine as a representative thiophosphate and a representative amine, respectively (Scheme 2). A mixed water/MeCN solvent system was employed where the reagents formed homogeneous solutions that we hoped to use for simple kinetic studies. When using the *N*-hydroxybenzotriazole system (leaving group p*K*_aH_ = 4.60 [5]), we performed several experiments at different pH values and at concentrations of ~0.02 M for all three components. These conditions represented a compromise between the lower concentrations required to maintain a homogeneous solution and the higher concentrations required to favour the bimolecular aminolysis and *S*-bridging-thiophosphate formation processes.

**Figure 1:**
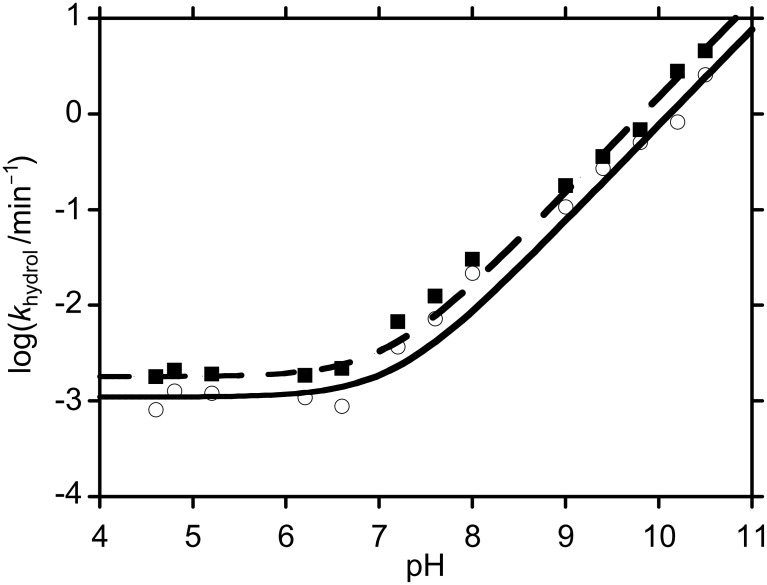
log *k*_hydrol_ vs pH for the hydrolysis *p*-nitrophenyl ester **7** (R = *p*NP) and *m*-nitrophenyl ester **7** (R = *m*NP) at 25 °C. Squares correspond to data for *p*-nitrophenyl ester **7** (R = *p*NP); circles correspond to data for *m*-nitrophenyl ester **7** (R = *m*NP). The dashed line corresponds to data fitting for *p*-nitrophenyl ester **7** (R = *p*NP) and the solid line corresponds to data fitting for *m*-nitrophenyl ester **7** (R = *m*NP).

Product mixtures were resolved by ion exchange chromatography (Supporting Information File 1), and product distributions were assessed by integration of the absorbance-retention time data (Supporting Information File 1, Figure 1). In the most favourable cases (pH 6.08 and 7.80), ~65% of the desired amide product was produced along with ~30% of the “carboxylate” arising through competing hydrolysis processes. Chromatographic separation proved time consuming and resolution of the products was poor, further illustrating the need to improve selectivity and avoid purification steps. Furthermore, the preparation of bromoacetyl-*N*-hydroxybenzotriazole **1** (R = Bt) proved troublesome, and the compound itself was not stable on storage. We next tried the *N*-hydroxysuccinimde system **1** (R = NHS) (leaving group p*K*_aH_ = 6.0 [6]) where we found that the desired amide product and *N*-hydroxysuccinimde co-eluted in the ion exchange chromatography, and, thus, we did not pursue the use of this system any further.

**Scheme 3:**
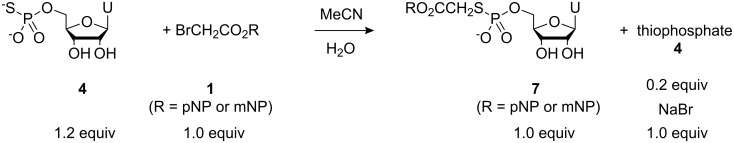
Ligation of 2-bromoacetic acid esters **1** (R = *p*NP or *m*NP) to thiophosphate **4**.

In order to gain an understanding of the aminolysis vs hydrolysis processes, we re-focused our studies towards activated bromoacetic acid phenyl ester systems that were more readily amenable to kinetic studies via UV–vis spectrophotometry. The 2-bromoacetic acid esters of *p*-nitro- and *m*-nitrophenol were allowed to react with thiophosphate **4** to generate *p*- and *m*-nitrophenyl 2-*S*-(5′-thiophosphoryluridine)acetates **7** (R = *p*NP) and **7** (R = *m*NP) (Scheme 3). In order to obtain simple kinetic data, it was essential to ensure that the nitrophenyl esters **7** (R = *p*NP) and **7** (R = *m*NP) were the only sources of the *p*- and *m*-nitrophenolate ions that were observed spectrophotometrically. With this in mind, thiophosphate **4** was used in excess to ensure complete, rapid consumption of the 2-bromoacetic acid-based phenyl esters **1** (R = *p*NP) and **1** (R = *m*NP) that could also give rise to *p*- and *m*-nitrophenolate ions during their hydrolyses. High concentrations of both thiophosphate **4** and 2-bromoacetic acid esters **1** (R = *p*NP) and **1** (R = *m*NP) were used in order to ensure that the S_N_2 reactions between these reagents occurred rapidly with minimal scope for hydrolysis of the esters during synthesis. Material containing 0.2 equiv of unreacted thiophosphate **4** and 1.0 equiv of NaBr by-product was then used for kinetic studies.

**Scheme 4:**
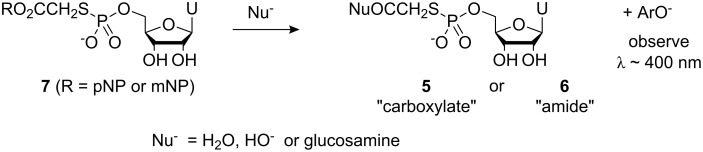
Displacement of *p*- or *m*-nitrophenolate ions from nitrophenyl esters **7** (R = *p*NP) and **7** (R = *m*NP).

Hydrolysis and aminolysis kinetic studies were carried out by observing spectrophotometrically the formation of *p*- or *m*-nitrophenolate ions arising from displacement of these ions from nitrophenyl esters **7** (R = *p*NP) and **7** (R = *m*NP) (Scheme 4).

Hydrolysis studies were performed in a range of buffers of differing pHs and strengths. The formation of *p*- and *m*-nitrophenolate ions was monitored at λ ~ 400 nm, and the spectrophotometric data showed simple exponential rises, confirming pseudo first order behaviour. We only found strong evidence for buffer catalysis when using HEPES and EPPS buffers, and even here, the effect was small (*k*_GB_ < 0.03 M^−1^min^−1^ in both cases). In these cases, recorded *k*_hydrol_ values correspond to extrapolation of experimental measurements over a range of different buffer concentrations, to zero buffer concentration. The fact that little general base assistance was observed is in line with observations of reactions between *p*-nitrophenyl acetate and other relatively non-basic amines [3]. Surprisingly, no evidence for general base catalysis was found with the more basic amine buffers (CHES and CAPS). Observed pseudo first order rate coefficients, *k*_hydrol_ (Table 1), were plotted as a function of pH (Figure 1), and the data were then fitted to a simple kinetic model for water- and hydroxide-promoted hydrolysis, namely,

[1]
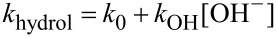


where

[2]
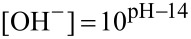


**Table 1:** Observed kinetic data for the hydrolysis of *p*-nitrophenyl ester **7** (R = *p*NP)^a^ and *m*-nitrophenyl ester **7** (R = *m*NP)^b^ at 25 °C.

pH	*k*_hydrol_ (min^−1^)^a^	*k*_hydrol_ (min^−1^)^b^

10.5	4.59	2.59
10.17	2.79	0.83
9.81	0.68	0.51
9.44	0.36	0.27
9.06	0.18	0.11
8.00	3.0 × 10^−2^	2.2 × 10^−2^
7.50	1.3 × 10^−2^	7.3 × 10^−3^
7.10	6.0 × 10^−3^	3.7 × 10^−3^
6.60	2.0 × 10^−3^	8.8 × 10^−4^
6.20	1.7 × 10^−3^	3.0 × 10^−3^
5.20	1.8 × 10^−3^	1.2 × 10^−3^
4.80	2.1 × 10^−3^	1.3 × 10^−3^
4.66	1.8 × 10^−3^	8.2 × 10^−4^

The pseudo first order rate coefficients, *k*_0_, for water-promoted hydrolyses of *p*-nitrophenyl ester **7** (R = *p*NP) and *m*-nitrophenyl ester **7** (R = *m*NP) were 1.8 × 10^−3^ min^−1^ and 1.1 × 10^−3^ min^−1^, respectively. The second order rate coefficients for hydroxide-promoted hydrolyses, *k*_OH_, for these species were 1.5 × 10^4^ M^−1^min^−1^ and 7.6 × 10^3^ M^−1^min^−1^, respectively.

**Figure 2:**
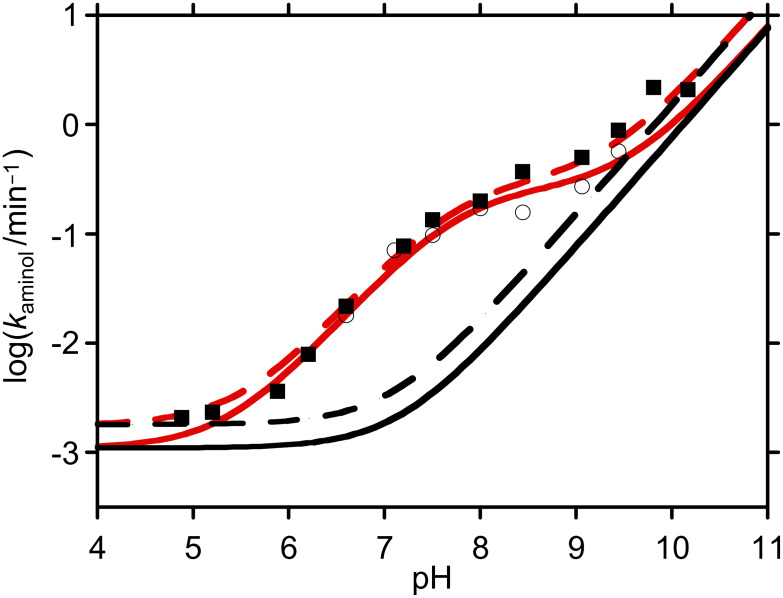
log *k*_aminol_ vs pH for the combined aminolysis and hydrolysis of *p*-nitrophenyl ester **7** (R = *p*NP) and *m*-nitrophenyl ester **7** (R = *m*NP) in the presence of 0.05 M D-glucosamine at 25 °C. Squares correspond to data for *p*-nitrophenyl ester **7** (R = *p*NP); circles correspond to data for *m*-nitrophenyl ester **7** (R = *m*NP). The dashed black line corresponds to data fitting for the hydrolysis of *p*-nitrophenyl ester **7** (R = *p*NP) and the solid black line corresponds to data fitting for the hydrolysis of *m*-nitrophenyl ester **7** (R = *m*NP) (Figure 1). The dashed red line corresponds to data fitting for the combined aminolysis and hydrolysis of *p*-nitrophenyl ester **7** (R = *p*NP); solid red line corresponds to data fitting for the combined aminolysis and hydrolysis of *m*-nitrophenyl ester **7** (R = *m*NP).

In order to assess the relative performance of *p*-nitrophenyl ester **7** (R = *p*NP) and *m*-nitrophenyl ester **7** (R = *m*NP) with respect to selective aminolysis in competition with hydrolysis processes, aminolysis studies were performed over a range of pHs. D-Glucosamine was chosen as a model amine system to investigate these properties because of its relatively low p*K*_aH_ of 7.75 [7]. Thus, even at relatively low pHs, a substantial proportion of the amine will be in its neutral, nucleophilic form. In addition, by structural analogy with ethanolamine, despite D-glucosamine’s relatively low basicity, it was still expected to display good nucleophilicity [8]. Observed pseudo first order rate coefficients, *k*_aminol_ (Table 2), were plotted as a function of pH (Figure 2), and the data were then fitted to the expression.

[3]
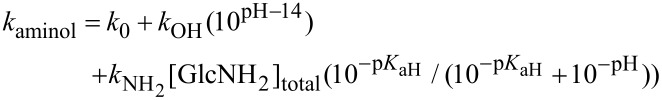


where fixed values for *k*_0_ and *k*_OH_ determined from the hydrolysis experiments, and a literature value for p*K*_aH_(GlcNH_2_) = 7.75 [7] was used.

**Table 2:** Observed kinetic data for the aqueous aminolysis and hydrolysis of *p*-nitrophenyl ester **7** (R = *p*NP)^a^ and *m*-nitrophenyl ester **7** (R = *m*NP)^b^ in the presence of 0.05 M D-glucosamine at 25 °C (n.d. = not determined).

pH	*k*_aminol_ (min^−1^)^a^	*k*_aminol_ (min^−1^)^b^

10.17	2.14	n.d.
9.81	2.19	n.d.
9.44	0.89	0.57
9.06	0.49	0.27
8.44	0.37	0.17
8.00	0.20	0.16
7.50	0.13	9.8 × 10^−2^
7.10	7.7 × 10^−2^	7.1 × 10^−2^
6.60	2.2 × 10^−2^	1.8 × 10^−2^
6.20	8.1 × 10^−3^	n.d.
5.88	3.7 × 10^−3^	n.d.
5.20	2.3 × 10^−3^	n.d.
4.88	2.1 × 10^−3^	n.d.

**Scheme 5:**
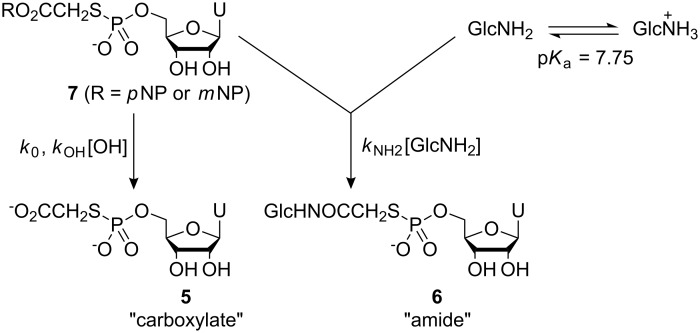
Kinetic model for competing hydrolysis and aminolysis processes of nitrophenyl esters **7** (R = *p*NP) and **7** (R = *m*NP) in the presence of D-glucosamine.

The second order rate coefficients for aminolysis by D-glucosamine, *k*_NH2_, for *p*-nitrophenyl ester **7** (R = *p*NP) and *m*-nitrophenyl ester **7** (R = *m*NP) were found to be 6.1 M^−1^min^−1^ and 5.1 M^−1^min^−1^, respectively. These results suggest that the *m*-nitrophenyl ester **7** (R = *m*NP) retains comparable reactivity towards aminolysis by D-glucosamine, whereas, it is less susceptible to attack by water and hydroxide ion than the *p*-nitrophenyl ester **7** (R = *p*NP). On this basis, the *m*-nitrophenyl ester **7** (R = *m*NP) would appear to offer better selectivity properties, and, thus, *m*-nitrophenyl 2-bromoacetate **1** (R = *m*NP) represents a potentially more effective heterobifunctional cross-linking agent than *p*-nitrophenyl 2-bromoacetate **1** (R = *p*NP). Our kinetic studies were performed under pseudo first order conditions in order to simplify kinetic analysis. However, with rate coefficient data in hand, we are able to make predictions on the relative selectivity of nitrophenyl esters **7** (R = *p*NP) and **7** (R = *m*NP) towards aminolysis over hydrolysis processes under more realistic conditions where activated ester and amine nucleophile are present at comparable concentrations. The concentrations of reagents in most synthetic laboratory reactions are usually ~0.01–0.05 M with one reagent being present in slight excess. Under standard conditions for bioconjugation processes, these concentrations are usually significantly lower and, consequently, lower yields of amide product are expected. With the more favourable synthetic applications in mind, we have used numerical integration techniques, in combination with the values for the rate coefficients *k*_0_, *k*_OH_ and *k*_NH2_, to predict the relative selectivities of nitrophenyl esters **7** (R = *p*NP) and **7** (R = *m*NP) towards aminolysis by D-glucosamine over hydrolysis (see experimental section for details of these calculations). Our kinetic model is described in Scheme 5.

**Figure 3:**
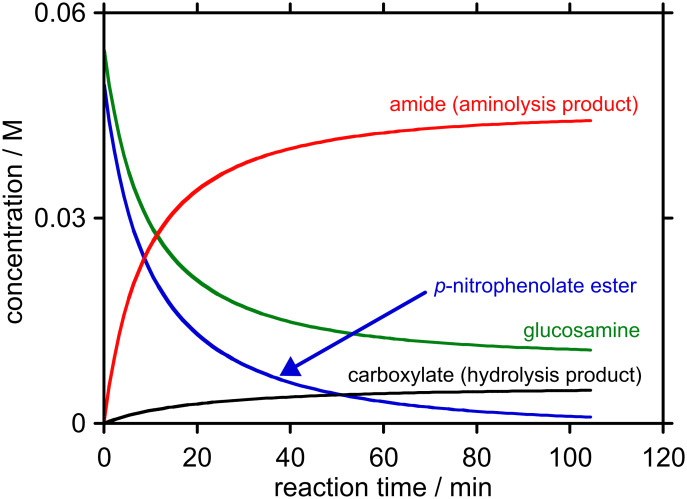
Predicted concentration-time profile for the reaction between starting concentrations of 0.05 M *p*-nitrophenyl ester **7** (R = *p*NP) and 0.055 M D-glucosamine at pH 7.41, 25 °C to 99% consumption of *p*-nitrophenyl ester **7** (R = *p*NP).

**Figure 4:**
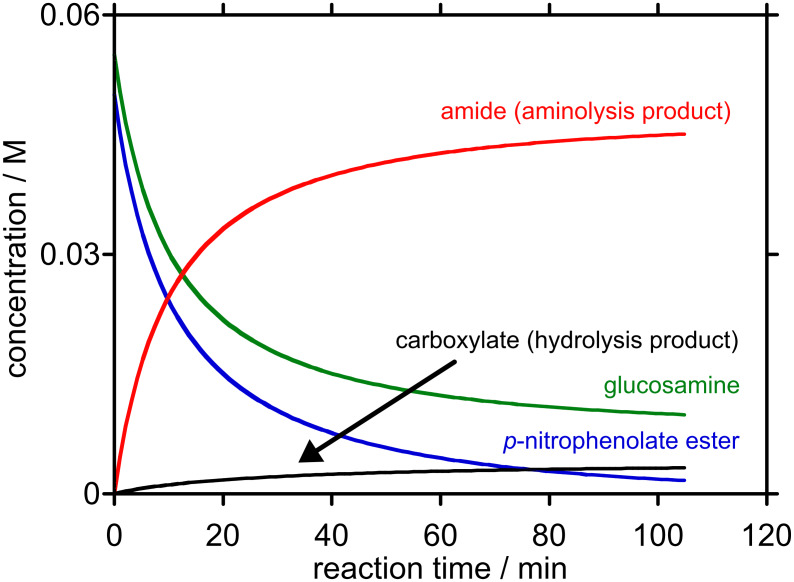
Predicted concentration-time profile for the reaction between starting concentrations of 0.05 M *m*-nitrophenyl ester **7** (R = *m*NP) and 0.055 M D-glucosamine at pH 7.46, 25 °C to 99% consumption of *m*-nitrophenyl ester **7** (R = *m*NP).

We chose to employ ester concentrations of 0.05 M and D-glucosamine concentration of 0.055 M that are expected to form homogeneous solutions that are amenable to simple kinetic modelling. Our predictive analyses were performed ~pH 7.4 based on King’s approach for predicting the optimal reaction pH for reactions between nucleophiles and reactive electrophiles [9]. The results from this exercise are plotted below (Figure 3 and Figure 4).

For the *p*-nitrophenyl ester **7** (R = *p*NP), pH_max_ was predicted to be 7.41, and 99% consumption of the ester was expected to take ~2 h. Under these conditions, 90% of the ester is expected to be converted to the amide. For the *m*-nitrophenyl ester **7** (R = *m*NP), pH_max_ was predicted to be 7.46, and 99% consumption of the ester was expected to take ~3 h. Under these conditions, 93% of the ester is expected to be converted into the amide. Clearly, these calculations show that selectivity towards aminolysis needs to be improved before this type of 2-*S*-(5′-thiophosphoryl)acetic acid ester or their bromoacetic acid ester progenitors can be used to provide product mixtures that do not require chromatographic purification. The *m*-nitrophenyl ester **7** (R = *m*NP) shows greater selectivity towards aminolysis than the *p*-nitrophenyl ester **7** (R = *p*NP), and, on this basis, we expect further improvements to be gained through increasing the p*K*_aH_ of the oxy-anionic leaving group.

By using simple Brønsted relationships, which have been repeatedly shown to be applicable to the hydrolyses and aminolyses of esters [3,10–11], we can estimate the effects that a change in the p*K*_aH_ of the ester leaving group is likely to have on the values of *k*_0_, *k*_OH_ and *k*_NH2_. Based on the values for *k*_0_, *k*_OH_ and *k*_NH2_ determined for the *p*- and *m*-nitrophenyl ester **7** (R = *p*NP) and **7** (R = *m*NP), and the known p*K*_a_ values for *p*- and *m*-nitrophenol, which are 7.14 and 8.35, respectively [12], the following Brønsted relationships can be considered:

[4]
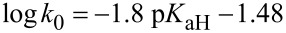


[5]
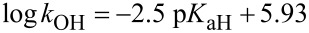


[6]
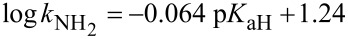


Studies on phenylacetic acid esters [3] and a range of 2-nitrophenyl 2-substituted acetates [11] show that simple Brønsted behaviour operates for the attack of amine nucleophiles, based on the p*K*_a_ of the conjugate acid of the amine (i.e. p*K*_aH_). Thus, additional kinetic data from the use of amines other than D-glucosamine could be used to assist predictions of *k*_NH2_ based on the nature of the amine nucleophile.

**Figure 5:**
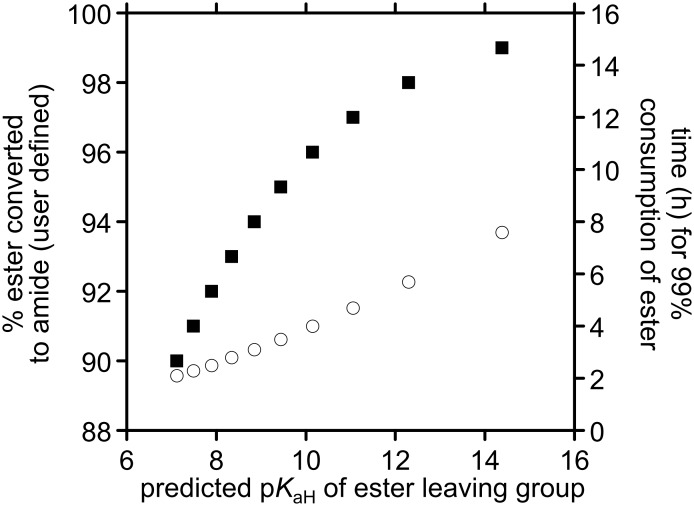
Predicted leaving group p*K*_aH_ values required for user-defined conversion levels of starting concentrations of 0.05 M 2-*S*-(5′-thiophosphoryluridine)acetic acid ester **7** and 0.055 M D-glucosamine to D-glucosamine amide **6** based on Brønsted extrapolations of *k*_0_, *k*_OH_ and *k*_NH2_. In each case, the calculations proceed to 99% consumption of ester. Squares correspond to % ester converted to amide in relation to p*K*_aH_ (left hand scale); circles correspond to time taken to attain 99% consumption of the ester with a leaving group of a given p*K*_aH_.

Using these Brønsted relationships, together with the starting reagent concentrations of 0.05 M 2-*S*-(5′-thiophosphoryluridine)acetic acid ester **7** and 0.055 M glucosamine, we have used numerical methods to predict the ester leaving group p*K*_aH_ values that are required to afford defined product distributions of aminolysis vs hydrolysis products. The solver function on Microsoft Excel™ was used to determine the p*K*_aH_ values of (oxy-anionic) leaving groups of 2-*S*-(5′-thiophosphoryluridine)acetic acid esters **7** that would be required to obtain given user-defined product distributions when 99% of the starting ester has been consumed. This process was performed for a range of user-defined product distributions over the range of 90–99% aminolysis product. The results from this process are presented in Figure 5.

These predictions rely on the validity of the Brønsted relationships across a broad p*K*_a_ range. At higher leaving group p*K*_a_ values, one or more of the relationships for *k*_0_, *k*_OH_ and *k*_NH2_ may break down. Esters with poorer leaving group (e.g. phenyl acetate) tend to exhibit general species catalysis in their hydrolyses and aminolysis, which will complicate the simple kinetic model that we have used, however, these processs are also expected to exhibit predictable Brønsted behaviour [3,10]. This change in behaviour corresponds to a change in rate determining step from the addition of nucleophile in the case of better leaving groups towards the expulsion of leaving groups in the case of poorer leaving groups. However, very good Brønsted behaviour for the *k*_OH_ term for acetyl ester hydrolysis has been observed across a very wide leaving group p*K*_a_ range [3], thus this term is expected to extrapolate well to higher leaving group p*K*_aH_ values. Furthermore, log *k*_0_ values usually correlate linearly with log *k*_OH_ values [10].

**Scheme 6:**
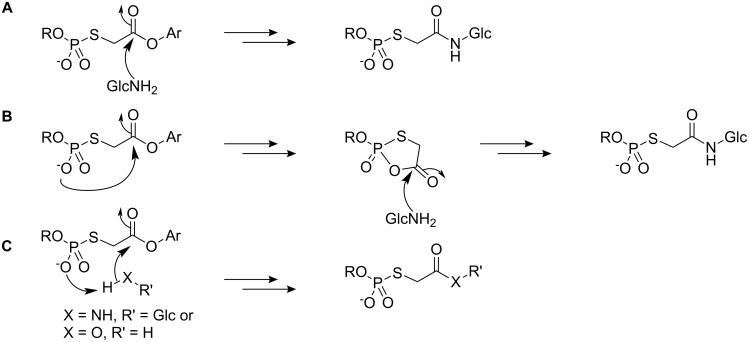
(A) Direct aminolysis of the ester carbonyl group; (B) intramolecular nucleophilic catalysis of ester cleavage followed by aminolysis of the mixed phosphoric-carboxylic anhydride; (C) intramolecular general base-assisted attack by amine or water.

In addition, our assumptions rely on a mechanism where the amine nucleophile attacks the ester carbonyl group and displaces the leaving group (Scheme 6A). Given the nature of the thiophosphate group in the 2-position of the ester, we may also consider the possibility of intramolecular nucleophilic catalysis where an oxygen atom of the phosphoryl group displaces the ester leaving group to form a cyclic mixed carboxylic-phosphoric acid anhydride (Scheme 6B) which is then attacked by amine. Alternatively, intramolecular general base catalysis of aminolysis and hydrolysis processes are also conceivable (Scheme 6C).

**Figure 6:**
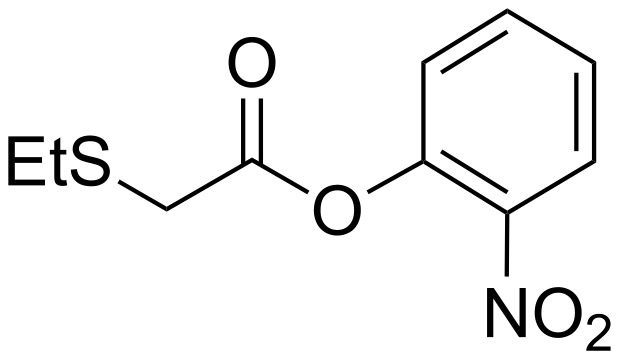
2-nitrophenyl 2-(ethylthio)acetate.

A comparison of the rate coefficients for the pH-independent hydrolysis of nitrophenyl esters **7** (R = *p*NP) and **7** (R = *m*NP) which were measured as *k*_0_ = 1.8 × 10^−3^ min^−1^ and 1.1 × 10^−3^ min^−1^, respectively with other 2-substituted acetates may provide insight at this point. Holmquist and Bruice have studied the hydrolysis kinetics of 2-nitrophenyl 2-(ethylthio)acetate at 30 °C [10], which is a thioether as opposed to the *S*-bridging-thiophosphates that form the basis of this paper (Figure 6).

Whilst we have used *p*-nitrophenyl- and *m*-nitrophenyl esters, and our kinetic studies were performed at 25 °C rather than 30 °C, some analogies with the *o*-nitrophenyl acetate system [10] may be drawn and conclusions may be inferred on the basis of similar leaving group properties of *o*-nitrophenol (p*K*_aH_ = 7.23 [12]). Rate coefficients for the pH-independent hydrolysis of 2-nitrophenyl 2-(ethylthio)acetate and 2-nitrophenyl acetate were reported in second order form as 1.32 × 10^−8^ M^−1^s^−1^ and 1.08 × 10^−7^ M^–1^s^−1^, respectively. In pseudo-first order form these coefficients translate to 4.36 × 10^−5^ min^–1^ and 3.56 × 10^−4^ min^−1^, respectively. When compared with *p*-nitrophenyl acetate, where *k*_0_ = 3.3 × 10^−5^ min^−1^ at 25 °C [13], *o*-nitrophenyl acetate shows comparable reactivity. On this basis, we would expect 4-nitrophenyl 2-substituted-acetates and 3-nitrophenyl 2-substituted-acetates to show similar reactivity trends to the 2-nitrophenyl 2-substituted-acetates reported by Holmquist and Bruice [10]. The 2-nitrophenyl 2-(ethylthio)acetate system with *k*_0_ = 3.56 × 10^−4^ min^−1^ shows comparable, if slightly lower, reactivity to the *p*- and *m*-nitrophenyl esters **7** (R = *p*NP) and **7** (R = *m*NP) with *k*_0_ = 1.8 × 10^−3^ min^−1^ and 1.1 × 10^−3^ min^−1^, respectively. Given these similar values, intramolecular catalysis of the displacement of the phenyl esters in our 2-*S*-thiophosphoryl acetate systems seems unlikely. When considering *k*_OH_ terms, *p*-nitrophenyl acetate and *o*-nitrophenyl acetate show similar reactivities with *k*_OH_ = 570 M^−1^min^−1^ and 1500 M^−1^min^−1^ (both at 25 °C), respectively, [10,14] which suggests that comparison between *o*-nitrophenyl and *p*- or *m*-nitrophenyl esters should be valid. Again, *p*- and *m*-nitrophenyl esters **7** (R = *p*NP) and **7** (R = *m*NP) with *k*_OH_ = 1.5 × 10^4^ M^−1^min^−1^ and 7.6 × 10^3^ M^−1^min^−1^, respectively, show comparable reactivity to 2-nitrophenyl 2-(ethylthio)acetate ester with *k*_OH_= 1.29 × 10^4^ M^−1^min^−1^. Holmquist and Bruice also performed aminolysis studies on 2-nitrophenyl 2-substituted-acetates [11]. Most salient to our work is a comparison with aminolysis using glycine ethyl ester, with the p*K*_aH_ of the amine being identical to that of D-glucosamine. Glycine ethyl ester displayed *k*_NH2_ = 1.1 M^−1^s^−1^ and 22.6 M^−1^s^−1^ when used in reactions with 2-nitrophenyl 2-(ethylthio)acetate and 2-nitrophenyl acetate, respectively. In our studies, using D-glucosamine, *k*_NH2_ values for *p*- and *m*-nitrophenyl esters **7** (R = *p*NP) and **7** (R = *m*NP) were found to be 6.1 M^−1^min^−1^ and 5.1 M^−1^min^−1^, respectively. Although marginally smaller than the value observed for 2-nitrophenyl 2-(ethylthio)acetate, both are similar. The same authors also found a linear correlation between log *k*_NH2_ and log *k*_0_ over four orders of magnitude in *k*_0_, which supports the idea that extrapolations of *k*_NH2_ can be made over a reasonably broad range of leaving group p*K*_aH_ values. Holmquist and Bruice observed that the linear correlation of the aminolysis rate coefficients *k*_NH2_ was also relatively insensitive to changes in hydrolysis rate coefficients *k*_0_ [11], which is in accord with our observations of relative insensitivity of *k*_NH2_ to leaving group p*K*_aH_.

## Conclusion

The esters *p*- and *m*-nitrophenyl esters **7** (R = *p*NP) and **7** (R = *m*NP) both display similar hydrolysis and aminolysis kinetics to 2-nitrophenyl 2-(ethylthio)acetate. The thiophosphoryl group appears to play a spectator role and does not contribute to catalysis in either the hydrolysis or aminolysis processes. Predictive studies suggest that both esters are likely to function as reasonably selective cross-linking agents, however, extrapolations, which appear reasonable in comparison to other acetate ester systems, suggest that an increase in leaving group p*K*_aH_ to ~12.4 should improve selectively so that chromatographic purification should be avoidable while retaining reasonable reaction times.

## Experimental

### 

#### Uridine-5′-monophosphorothioate **4**

Dry uridine (1 g, 4.1 mmol) was dissolved in freshly distilled triethylphosphate (10 mL) by heating at 50 °C under a nitrogen atmosphere. The flask was then transferred to an ice bath. Ice-cooled 2,6-dimethylpyridine (1.4 mL, 12.3 mmol) and ice-cooled thiophosphoryl chloride (0.75 ml, 7.4 mmol) were added sequentially to the solution and the mixture was stirred for 2 h at 4 °C under a nitrogen atmosphere. The mixture was allowed to warm to room temperature and then poured into petroleum ether (bp 40–60, 300 mL). The white precipitate was allowed to settle, the solvents were decanted and the residual white solid was washed with petroleum ether (2 × 100 mL). Iced water (50 mL) was then added to the solid, and the mixture stirred at 4 °C for 2 h. The solution was adjusted to pH 8 by the addition of potassium hydroxide while warming to room temperature. The solution was extracted successively with diethyl ether (2 × 100 mL) and petroleum ether (bp 40–60, 2 × 100 mL), and the aqueous layer lyophilised for storage. The crude thiophosphate **4** was dissolved in equilibrating buffer (50 mM triethylammonium bicarbonate solution (TEAB) [15], pH 7.6) and loaded using a 50 mL superloop onto a DEAE Sepharose FF column (500 ml bed volume, in a 30 × 5 cm column). Anion exchange chromatography was performed using a linear gradient of triethylammonium bicarbonate solution (50–600 mM) pH 7.6 [15], over 2 h at a flow rate of 30 mL/min using an Äkta Plus chromatography system. Fractions containing the desired product, eluted between 200 and 300 mM of triethylammonium bicarbonate, were combined and lyophilised to give uridine-5′-monophosphorothioate **4** as the bis(triethylammonium) salt. Triethylammonium ions were exchanged for sodium ions by mixing a solution of the bis(triethylammonium) thiophosphate **4** salt (0.6 g from several chromatographic runs) in water (2.5 mL), and a solution of sodium iodide (0.6 g, 4 mmol) in acetone (12.5 mL). Methanol (2.5 mL) and diethyl ether (2.5 mL) were added to assist precipitation. The mixture was centrifuged at 4500 rpm for 10 min and the supernatant liquid decanted. After washing with acetone (2 × 25 mL), the resulting sticky, amorphous pellet was re-dissolved in water and lyophilised to afford a white powder of the disodium salt of uridine-5′-monophosphorothioate **4** (0.3 g, 37%). (Found C, 24.98; H, 3.76; N, 6.38. C_9_H_11_O_8_N_2_PSNa_2_ requires C, 24.67; H, 3.91; N, 6.39%); ν_max_ (KBr disk)/cm^−1^ 3220 (OH), 1690 (CO imide), 1270 (PO), 620 (PS); δ_H_ (500 MHz; D_2_O) 7.99 (1H, d, *J* 8.0, 6-C*H*), 5.82 (1H, d, *J* 5.4, 1′-C*H*), 5.79 (1H, d, *J* 8.1, 5-C*H*), 4.24 (1H, t, *J* 5.1, 2′-C*H*), 4.20 (1H, t, *J* 4.5, 3′-C*H*), 4.08–4.12 (1 H, m, 4′-C*H*), 3.82–3.93 (2 H, m, 5′-C*H*); δ_P_ (160 MHz, D_2_O) 44.2; δ_C_ (125 MHz; D_2_O) 167.3 (4-*C*=O), 152.7 (2-*C*=O), 142.3 (6-*C*H), 102.8 (5-*C*H), 88.3 (1′-*C*H), 84.2 (4′-*C*HP), 74.1 (2′-*C*H), 70.3 (3′-*C*H), 63.6 (5′-*C*H_2_); *m/z* (ES^–^) 339.0059 (M – H. C_9_H_12_N_2_O_8_PS requires 339.0057).

#### *p*-Nitrophenyl 2-bromoacetate **1** (R = *p*NP)

A solution of pyridine (0.35 mL, 4.3 mmol) in dry DCM (5 mL) was added dropwise to a stirred solution of bromoacetyl bromide (0.38 mL, 4.3 mmol) in dry DCM (5 mL) cooled in an ice bath. Following the careful addition of *p-*nitrophenol (0.6 g, 4.3 mmol), the reaction mixture was stirred for 1 h. Saturated sodium bicarbonate solution (5 mL) was added, the layers were separated, and the organic layer was washed successively with water (2 × 5 mL), hydrochloric acid (0.1 M, 3 × 5 mL) and saturated sodium chloride solution (5 mL). The organic phase was then dried over anhydrous magnesium sulphate and the solvent removed under reduced pressure to give the *p*-nitrophenyl ester (0.752 g, 67%); mp = 72–75 °C (dec); (Found C, 36.94; H, 2.31; N, 5.17. C_7_H_6_BrNO_4_ requires C, 36.92; H, 2.31; N, 5.38%); ν_max_ (KBr disc)/cm^−1^ 3110–2963 (CH), 2847 (CH_2_), 1770 (ester CO); *δ*_H_ (500 MHz; CDCl_3_) 8.31 (2 H, d, *J* 9.0, C*H*CNO_2_), 7.34 (2 H, d, *J* 9.3, C*H*CO), 4.08 (2 H, s, CO*CH_2_*Br); *δ*_C_ (125 MHz; CDCl_3_) 165.2 (*C*=O), 155.1 (*C*O), 145.9 (*C*NO_2_), 125.6 (NO_2_C*C*H), 122.4 (*C*HCO), 25.3 (*C*H_2_Br); *m/z* (EI) 258.9 and 260.9.

#### *m*-Nitrophenyl 2-bromoacetate **1** (R = *m*NP)

The procedure for *p*-nitrophenyl 2-bromoacetate **1** (R = *p*NP) was followed except *p*-nitrophenol was replaced by *m*-nitrophenol (0.6 g, 4.3 mmol). After work up, the *m*-nitrophenyl ester was obtained (0.697 g, 62%); mp = 50–53 °C (dec); (Found C, 36.96; H, 2.31; N, 5.39. C_7_H_6_BrNO_4_ requires C, 36.92; H, 2.31; N, 5.38%); ν_max_ (KBr disc)/cm^−1^ 3116–3011 (CH), 2864 (CH_2_), 1777 (ester CO); *δ*_H_ (500 MHz; CDCl_3_) 8.16 (1 H, d, *J* 8.8, 4-C*H*), 8.05 (1 H, s, 4-C*H* ), 7.63 (1 H, t, *J* 8.1, 5-C*H*), 7.53 (1 H, t, *J* 8.2, 6-C*H*), 4.10 (2 H, s, *CH_2_*Br); *δ*_C_ (125 MHz; CDCl_3_) 165.5 (*C*=O), 150.7 (*C*O), 149.0 (*C*NO_2_), 130.6 (5-*C*H), 127.8 (6-*C*H), 121.6 (4-*C*H), 117.3 (2-*C*H), 25.2 (*C*H_2_Br); *m/z* (EI) 258.9 and 260.9.

#### *p*- and *m*-Nitrophenyl 2-*S*-(5′-thiophosphoryluridine)acetates **7** (R = *p*NP) and **7** (R = *m*NP) for kinetic studies

The disodium salt of uridine-5′-monophosphorothioate **4** (1 eq, 20 mg, 58.8 μmol) was dissolved in deionised water (0.5 mL) and a solution of *p*- or *m*-nitrophenyl 2-bromoacetate **1** (R = *p*NP) or **1** (R = *m*NP) (0.8 eq, 12 mg, 46 μmol) in acetonitrile (0.5 mL) added. The mixture was stirred for one minute then rapidly frozen in liquid nitrogen followed by lyophilisation to give a light yellow solid of intermediate, *p*- or *m*-nitrophenyl 2-*S*-(5′-thiophosphoryluridine)acetate **7** (R = *p*NP) or **7** (R = *m*NP) (~80% purity by ^1^H NMR spectroscopy, contaminated with excess uridine-5′-monophosphorothioate **4**). Stock solutions were prepared by dissolving crude *p*- or *m*-nitrophenyl 2-*S*-(5′-thiophosphoryluridine)acetate **7** (R = *p*NP) or **7** (R = *m*NP) (10 mg) in deionised water (3 mL), and this was divided into portions that were frozen in liquid nitrogen. Owing to the instability and crude nature of the nitrophenyl 2-*S*-(5′-thiophosphoryluridine)acetate esters in aqueous solution, only ^1^H NMR and ES^−^ analyses were performed. Data for *p*-nitrophenyl 2-*S*-(5′-thiophosphoryluridine)acetate **7** (R = *p*NP) *δ*_H_ (500 MHz; D_2_O) 8.11 (2 H, d, *J* 9.2, C*H*CNO_2_), 7.55 (1 H, d, *J* 8.2, 5-C*H*), 7.24 (2H, d, *J* 9.2, *CH*CO), 5.72 (1H, d, *J* 3.9, 1′-C*H*), 5.55 (1H, d, *J* 8.2, 6-*CH*), 4.32–3.91 (5 H, m, 2′–5′-C*H*), 3.7 (2 H, d, *J* 15.4, S*CH_2_*); *δ*_P_ (80 MHz; D_2_O) 19.2; *m/z* (ES^−^) 518.0 (M–H for UMPS-CH_2_CO_2_-*p*-C_6_H_4_NO_2_), 379.1 (M–H for the cyclic hydrolysis product, UMPSCH_2_CO_2_^−^).

#### Hydrolysis studies on *p*- and *m*-nitrophenyl 2-*S*-(5′-thiophosphoryluridine)acetates **7** (R = *p*NP) and **7** (R = *m*NP)

Kinetic measurements were performed by mixing stock solution of the ester (25 μL) with buffer (1.5 mL) to give ~0.1 mM final concentration of ester in the cuvette. The cuvette was inserted into a thermostated (25 °C) compartment of the UV–vis spectrophotometer and the increase in absorbance of *p*- or *m*-nitrophenolate monitored at λ ~ 400 nm. The kinetic data were fitted to the function *A*_t_ = *A*_0_+*A*_∞_(1–e^–^*^k^*^obs t^), and showed clean first order behaviour with observed rate constants *k*_0_.

#### Aminolysis studies on *p*- and *m*-nitrophenyl 2-*S*-(5′-thiophosphoryluridine)acetates **7** (R = *p*NP) and **7** (R = *m*NP)

D-Glucosamine solution (1.24 M, 60 μL) was mixed with buffer (0.5 M, 1.44 mL) to generate a solution (1.5 mL) with 50 mM final concentration of D-glucosamine in the cuvette. Stock solution of ester was then added, and the kinetics were monitored and analysed as described above.

### Buffer preparation for kinetic studies

Buffers were prepared using CAPS (pH 10.5 and 10.17), CHES (pH 9.81, 9.44 and 9.06), EPPS (pH 8.44 and 8.00), HEPES (pH 7.50 and 7.10), MES (pH 6.60, 6.00 and 5.88) and acetate (pH 4.80 and 4.66) systems where pHs were adjusted by the addition of hydrochloric acid or hydroxide solutions. Buffer strengths of 0.05, 0.1, 0.2, 0.3 and 0.5 M were used to check for general species-promoted hydrolysis. All aminolysis studies were performed using 0.5 M buffers in the presence of 0.05 M D-glucosamine.

### Data analysis and kinetic predictions

Kinetic data were analysed using Kaleidagraph™. Kinetic predictions were based on Brønsted relationships between observed rate coefficients *k*_0_, *k*_OH_ and *k*_NH2_ and the p*K*_aH_ of the leaving group. Minimisations were performed using the Solver function in Microsoft Excel™. A spreadsheet was constructed where the reaction was modelled over 1500 time points that were equally spaced to cover 99% consumption of the ester. King’s approach towards estimating pH_max_ [9] was adopted in order to ensure that all product distribution estimations were performed at the optimum pH. Minimisations centred on predicting the values of *k*_0_, *k*_OH_ and *k*_NH2_ that would lead to a given proportion of the desired amide product, subject to the Brønsted relationship constraints and King’s pH_max_ estimation. The Excel™ spreadsheet is available as Supporting Information File 2.

## Supporting Information

Supporting information features a PDF document presenting details of studies using the bromoacetyl-OBt system **1** (R = Bt) and bromoacetyl-NHS system **1** (R = NHS), and associated experimental details, as well as Excel™ spreadsheets for the prediction of product outcomes and the optimisation of leaving group p*K*_aH_ values of 2-*S*-thiophosphate esters with reference to controlling aminolysis over hydrolysis.

File 1 Synthesis and application of bromoacetyl-OBt and NHS systems

File 2 Prediction of product distribution using *p*-nitrophenyl ester **7** (R = *p*NP)

File 3 Prediction of product distribution using *m*-nitrophenyl ester **7** (R = *m*NP)

File 4 Optimisation of leaving group p*K*_aH_ values of 2-*S*-thiophosphate esters
