# Supplementary material for: Kinetic studies and predictions on the hydrolysis and aminolysis of esters of 2-S-phosphorylacetates
Source: Beilstein J Org Chem. 2010 Aug 16;6:732–41. doi: 10.3762/bjoc.6.87 (PMC2956570; doi:10.3762/bjoc.6.87)
Supplement: File 1 — Synthesis and application of bromoacetyl-OBt and NHS systems [file Beilstein_J_Org_Chem-06-732-s001.pdf]

Supporting Information File 1

for

Kinetic studies and predictions on the hydrolysis and aminolysis of esters of 2-*S*-phosphorylacetates

Milena Trmčić and David R. W. Hodgson*

Address: Centre for Bioactive Chemistry, Department of Chemistry, Durham University, Science Laboratories, South Road, Durham, DH1 3LE, United Kingdom

Email: Milena Trmčić - milatrmcic@yahoo.com; David R. W. Hodgson* - d.r.w.hodgson@durham.ac.uk

* Corresponding author

Synthesis and application of bromoacetyl-OBt and NHS systems

Bromoacetyl-*N*-hydroxybenzotriazole 1 (R = Bt)

A solution of pyridine (0.71 mL, 8.7 mmol) in dry DCM (10 mL) was added dropwise to a stirred solution of bromoacetyl bromide (0.76 mL, 8.7 mmol) in dry DCM (10 mL) cooled in an ice bath. Following the careful addition of HOBt (0.6 g, 8.7 mmol), the reaction mixture was stirred for 1 h. The work-up consisted of successive quick washes of the organic phase with water (2 × 10 mL), hydrochloric acid (0.1 M, 3 × 10 mL) and saturated sodium chloride solution (10 mL). The organic extracts were then dried over anhydrous magnesium sulphate and the solvent was removed under reduced pressure to give the crude product as a dark yellow solid (0.83 g, 75%). The purity was estimated by ^{1}H NMR spectroscopy (70%). δ_H (500 MHz; $CDCl_3$) 8.34 (1 H, d, *J* 8.5, 7-C*H*), 7.96 (1 H, d, *J* 8.4, 4-C*H*), 7.77 (1 H, t, *J* 7.8, 6-C*H*), 7.55 (1 H, t, *J*

7.7, 5-C*H*), 4.53 (2 H, s, CH_2Br); δ_C (126 MHz; D_2O) 162.6 (*C*=O), 138.7 (quaternary), 133.5 (6-*C*H), 131.4 (quaternary), 127.5 (5-*C*H), 116.1 (7-*C*H), 115.7 (4-*C*H), 25.6 (CH_2Br). This material proved to be unstable even when stored at −20 °C.

Bromoacetyl-*N*-hydroxysuccinimide 1 (R = NHS)

The same procedure as for the synthesis of bromoacetyl-*N*-hydroxybenzyltriazole was applied with *N*-hydroxysuccinimide (1 g, 8.7 mmol) in place of HOBt, and saturated sodium bicarbonate solution was used in place of water during the first stage of the reaction work up. The organic extracts were then dried over anhydrous magnesium sulphate and the solvent was removed under reduced pressure to give the crude product (1.52 g, 74%). The purity was estimated by ^{1}H NMR spectroscopy (100%). mp = 97–102 °C (dec); ν_{max} (KBr disk)/cm^{-1} 2937-3056 (CH_2), 1750–1811 (CO); δ_H (400 MHz; $CDCl_3$) 4.09 (2 H, s, C*H*$_2$Br), 2.79 (4 H, s, C*H*$_2$C*H*$_2$); δ_C (100 MHz; $CDCl_3$) 168.5 (O*C*=O), 163.1 (N*C*=O), 25.6 (*C*H_2Br) 21.2 (*C*H_2*C*H_2); *m/z* (EI) 234.9 and 236.9.

Preparative scale reaction between bromoacetyl-*N*-hydroxybenzotriazole and glucosamine for the preparation of authentic samples of "amide" and "carboxylate" products.

A solution of crude bromoacetyl-*N*-hydroxybenzotriazole 1 (R = Bt) (23.1 mg) in dry acetonitrile (3 mL) was mixed with an aqueous solution (2 mL) of the disoduim salt of uridine-5'-monophosphorothioate **4** (30 mg, 0.075 mmol) and D-glucosamine (19.5 mg, 0.086 mmol). Aqueous sodium hydroxide (1 M) was added dropwise until the pH reached ~9 (pH paper). The mixture was then stirred for 12 h followed by the removal of the acetonitrile under reduced pressure leaving the product mixture in a basic aqueous solution. The aqueous solution of crude sample was loaded onto a pre-equilibrated anion exchange DEAE Sephadex FF column (50 ml, 10 × 3 cm) and eluted using TEAB buffer, pH 7.6 linear gradient (50–400 mM). Fractions eluted between 70 and 100 mM (peak 2) of TEAB buffer and between 200 and 230 mM of TEAB (peak 5) were pooled separately and lyophilised. The triethylammonium salts of the products from peaks 2 and 5 were dissolved separately in water (5 mL) and eluted with water on a Dowex® 50W × 2, 200-400 Na^+ form (50 mL, 30 × 2 mm, 3 mL/min) column. The fractions containing product, detected by monitoring A_{280} were collected and lyophilised.

Sodium salt of the "amide" product 6

The purity of the compound was estimated by ^{31}P and ^{1}H NMR spectroscopy (both 100%) and the anomer distribution was found to be *α* 55% and *β* 45%. ν_{max} (KBr disc)/cm^{-1} 3330 (OH), 2940 (NH imide), 1690 (CO imide), 1230 (PO), 570 (PSR); δ_H (500 MHz; D_2O) 7.74 (1 H (α + β), d, *J* 8.1, 5-C*H*), 5.80 (1 H (α + β), d, *J* 5.1, 1´-C*H*), 5.78 (1 H (α + β), d, *J* 8.1, 6-C*H*), 5.03 (1H, d, *J* 3.5, α-C*H*), 4.5 (1H, d, *J* 8.3, β-C*H*), 4.21–4.10 (3 H (α + β), m, 2´-, 3´- and 4´-C*H*), 3.76–3.28 (8 H (α + β), m, C*H2*S and GlcNH), 4.09–3.94 (2 H (α + β), m, CHC*H2*OP); δ_P (160 MHz, D_2O) 18.3; δ_C (500 MHz; D_2O) 174.3 (2 × d, $^{3}J_{C\text{-}P}$ 2.2 and 4.1, α and β-*C*=OCH$_2$S), 166.3 ((α+β) 4-*C*=O), 151.8 ((α + β) 2-*C*=O), 141.7 ((α + β) 6-*C*H), 102.7 ((α + β) 5-*C*H), 94.8 (β-*C*H), 90.9 (α-*C*H), 88.9 (2 × s, (α + β) 1´-*C*H), 82.9 (m, (α+β) 4'-*C*H), 76.1 (β-CH$_2$*C*HO), 73.8 (α-CH$_2$*C*HO), 73.8 (2 × s, (α + β) 2´-*C*H), 71.7–69.8 (4 × s, (α + β)-GlcNH), 69.7 (2 × s, (α + β) 3´-*C*H), 65.0 (m, (α + β) 5´-*C*H$_2$), 60.8 (2 × s, (α + β) *C*H$_2$OH), 57.5 (β-*C*HNH), 54.6 (α-*C*HNH), 33.4 (2 × s, (α+β) *C*H$_2$S); *m/z* (ES$^-$) 550.0795 (M–H. $C_{17}H_{25}N_3O_{14}PS$ requires 558.0800).

Sodium salt of the "carboxylate" by-product 5

ν_{max} (KBr disc)/cm^{-1} 3250 (OH), 2940 (NH imide), 1690 (CO amide), 1260 (PO), 1210 (PO), 570 (PSR); δ_H (400 MHz; D_2O) 7.74 (1 H, d, *J* 8.1, 5-C*H*), 5.80 (1 H, d, *J* 5.0, 1´-C*H*), 5.78 (1 H, d, *J* 8.1, 6-C*H*), 4.17 (1 H, t, *J* 5.1, 2´-C*H*), 4.14 (1 H, t, *J* 5.0, 3´-C*H*), 4.10–4.08 (1 H, 4´-C*H*), 4.05–3.91 (2 H, m, 5´-C*H*$_2$), 3.38 (2 H, d, *J* 14.5, C*H*$_2$S); δ_P (160 MHz, D_2O) 19.8; δ_C (125 MHz; D_2O) 174.0 (d, $^{3}J_{C\text{-}P}$ 3.9, *C*=OCH$_2$S), 166.3 (4-*C*=O), 151.9 (2-*C*=O), 141.7 (6-*C*H), 102.7 (5-*C*H), 88.7 (1´-*C*H), 83.0 (d, $^{3}J_{C\text{-}P}$ 9.0, 4´-*C*H), 73.8 (2´-*C*H), 69.7 (3'-*C*H), 65.0 (d, $^{3}J_{C\text{-}P}$ 6.3, 5´-*C*H$_2$), 32.1 (*C*H$_2$S); *m/z* (ES$^-$) 397.0111 (M–H. $C_{11}H_{14}N_2O_{10}PS$ requires 397.0112).

Investigation of the effects of pH on the cross-linking between UMPS and D-glucosamine using bromoacetyl-*N*-hydroxybenzotriazole 1 (R = Bt)

Reactions were performed by preparing a range of reaction mixtures with different pH values. Solutions of thiophosphate **4** (20 mg, 0.05 mmol) in water (0.1 mL) and D-glucosamine hydrochloride (13.6 mg, 0.06 mmol) in water (0.2 mL) were mixed in each tube. Crude bromoacetyl-*N*-hydroxybenzotriazole (13.5 mg) was dissolved in acetonitrile (0.4 mL), previously dried over molecular sieves, and added to each of

the aqueous solutions. Sodium hydroxide solution (1 M aqueous solution) was then added according to table 7.9, to adjust to a given pH that was measured by a pH meter. Each mixture was stirred for 12 h, then the acetonitrile was evaporated under reduced pressure and the samples were lyophilised prior to being subjected to analytical anion exchange chromatography.

Table 1: Number of equivalents of sodium hydroxide solution employed in each experiment and measured pHs of the resulting mixtures.

**Reaction**	**I**	**II**	**III**	**IV**	**V**	**VI**	**VII**
**Number of equivalents of of 1 M $NaOH_{(aq)}$**	0.5	0.75	1	1.25	1.5	1.75	2.25
**Measured pH (H_2O, MeCN)**	4.8	6.1	7.8	9.2	10.8	12.7	13.7

Analytical anion exchange chromatography

Samples were prepared by dissolving lyophilised solid (1 mg) in TEAB solution (0.05 M, 0.5 mL) and injecting the sample on to a pre-equilibrated analytical (1 mL) anion exchange column (DEAE Sepharose FF). A linear gradient elution profile of 50–120 mM TEAB buffer, pH 7.6, with a flow rate of 1 mL/min was performed over the course of 30 min. The chromatograms are presented in Figure 1.

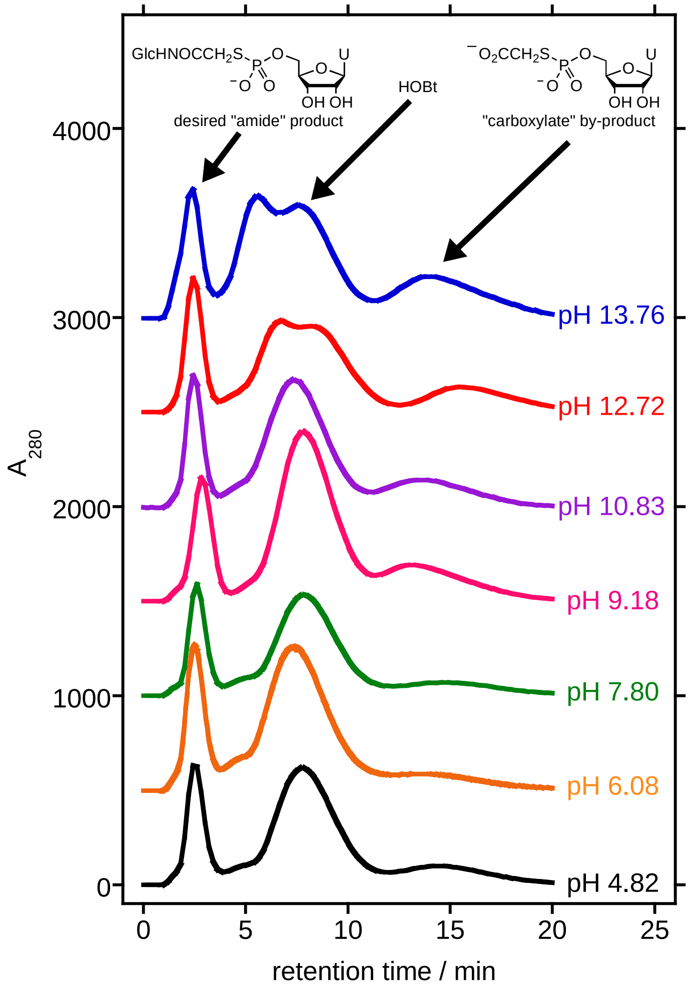

Figure 1: Anion exchange chromatograms of reaction mixtures of bromoacetyl-*N*-hydroxybenzotriazole, UMPS and D-glucosamine where reactions were performed under different pH conditions at room temperature for 12 h.
